# Discharge against Medical Advice at Neonatal Intensive Care Unit in Gujarat, India

**DOI:** 10.1155/2016/1897039

**Published:** 2016-11-24

**Authors:** Bhanu Devpura, Pranav Bhadesia, Somashekhar Nimbalkar, Sandeep Desai, Ajay Phatak

**Affiliations:** ^1^Department of Pediatrics, Pramukhswami Medical College, Karamsad, Anand, Gujarat 388325, India; ^2^Central Research Services, Charutar Arogya Mandal, Karamsad, Anand, Gujarat 388325, India; ^3^Charutar Arogya Mandal, Karamsad, Anand, Gujarat 388325, India

## Abstract

*Objective*. We explored reasons for discharged against medical advice (DAMA) of neonates from a neonatal intensive care unit (NICU) through in-depth interviews of the parents/guardians.* Methods*. Of 456 babies admitted to NICU during April 2014 to March 2015, 116 babies were DAMA. Parents of randomly selected 50 babies of these 116, residing within 50 kilometers, were approached for in-depth interviews at their homes. Audio recordings were done and manually transcribed, analyzed in detail to explore common threads leading to DAMA. Basic demographic information of the newborns was retrieved from hospital records.* Results*. The prevalence of DAMA was 25.4%. Of 50 parents approached, 41 in-depth interviews were completed. Nonaffordability (38.6%), no improvement (14.6%), poor prognosis (12%), and inappropriate behavior of the patient relation office personnel (10.6%) were major factors contributing to DAMA. Parents of 6.6% neonates wanted guarantee of survival and 5.3% parents reported poor behavior of nurses. No gender bias was observed related to DAMA. One-third of neonates (34.1%) were DAMA on first day of admission.* Conclusions*. The issue of DAMA needs attention. Besides nonaffordability and clinical characteristics of the baby, communication (breaking bad news, counseling, etc.) and lack of adequate infrastructure for relatives emerged as modifiable factors leading to DAMA.

## 1. Introduction 

The twentieth century witnessed transformation in human health. Advent of faster and miniature computers had cascading effect leading to exponential growth in technology and modern medicine that led to better health care in all age groups and across gender. The darker legacy of these advancements is the staggering cost of healthcare.

Significant improvements in maternal and child care indices were noted in India especially in last decade though India missed Millennium Development Goals (MDGs) 4 and 5 targets. India, with about 0.76 million neonatal deaths per year, recorded maximum neonatal deaths in the world in 2012 [1]. Further the decline in Neonatal Mortality Rate (NMR) is much slower than the decline in Infant Mortality Rate (IMR) and under 5-mortality rate resulting in appreciable share of neonatal mortality in overall childhood mortality [2]. Current figures reveal that neonatal mortality contributed to 70% of infant mortality and 57% of under-five mortality [3].

While there is a paradigm shift from hospital care to home care in developed economies partly to reduce healthcare costs, the developing economies look forward to institutional care for better health outcomes. To address the huge neonatal morality, facility based newborn care was introduced in India which was quite successful with about 90% survival rate in 2012-13 [2].

The advantages of institutional care are dampened by many factors including Healthcare Acquired Infections (HAIs) and withdrawal from treatment by the patients. Discharge against Medical Advice (DAMA) happens when a patient (or the parents or caregivers, in the case of a newborn) decides the timing of the discharge without a treating doctor's approval. DAMA not only raises clinical, ethical, and legal issues for the treating physician [4] but also leads to adverse health outcomes thereby burdening the health system even more [5]. There have been few attempts by developing economies to document the extent of the problem as well as the reasons behind DAMA in pediatric population [6–11] and neonates [12, 13]. The studies in pediatric populations indicated that the problem is more prevalent in neonates. This has serious consequences considering limited physiological reserve of the neonates. These studies also indicated that reasons for DAMA vary according to settings, culture, and other factors.

Understanding the sociocultural aspects of DAMA is very important for a country like India which is investing a lot to reduce neonatal mortality through combination of Home Based Newborn Care (HBNC) and facility based newborn care (FBNC). This study aims to identify the reasons for DAMA from the neonatal intensive care unit (NICU) of a tertiary care hospital through in-depth interviews of the parents/guardians of the babies who were discharged against medical advice.

## 2. Methodology

The study was conducted at Shree Krishna Hospital, a rural tertiary care teaching hospital in Gujarat State of India. Parents of babies who were admitted to NICU during April 2014 and March 2015 constituted the sampling frame.

The NICU has a capacity of 22 beds and provides level III neonatal care. It is managed by 22 nurses who work in shift duty and 3 dedicated consultants. Four residents and 2 fellows are posted in NICU at any given time. The bed occupancy varies from 70% to 80% with average nurse to patient ratio of 1 : 2.

Shree Krishna Hospital is managed by Charutar Arogya Mandal, a nonprofit trust founded by the late HM Patel with a philanthropic vision. The hospital offers quality treatment at affordable cost to public at large. It offers huge discount to the underprivileged through a targeted approach. Typically, any child requiring critical care is offered 75% discount (excluding pharmacy) on the total bill if the parents are able to produce the below poverty line (BPL) card. Daily counseling about the child's condition is done by consultants/fellows whereas financial counseling is done by patient relation office (PRO) staff dedicated to NICU. Full/partial extra discount is offered to a child with/without BPL card through an elaborate system considering prognosis and paying capacity of the parents. All efforts are made by PRO staff to ensure that no child is denied treatment due to parent's inability to pay. After all possible efforts, if parents still want discharge against medical advice, a written consent is obtained from the parents. Albeit not a common practice in India, the parents are provided with a discharge summary to help them take informed decision.

Out of 456 babies admitted in NICU from April 2014 to March 2015, 116 (25.4%) babies were discharged against medical advice. Fifty of these 116 babies residing within 50 kilometers of the hospital were selected randomly using software, namely, WINPEPI. However, the purpose of randomization was just to ensure that the interviewers do not select only villages in close vicinity of the hospital.

Parents of these 50 selected babies were contacted and in-depth interview was conducted at their homes based on an interview guide (Appendix). As per the legal system, one of the parents has to be present during the DAMA process and most of the times they are the primary caretakers. A team of three persons (one dedicated member from the investigators with any two pediatric postgraduates from the hospital) participated in conducting the interview. The dedicated person conducted all the interviews while the postgraduates wrote down the necessary details. Audio was recorded if consent was given for the same. The parents (both mother and father) were invited to participate and were provided with the contact details of the interviewers so that they could ask for deletion of the any part of audio/interview during the study. In 13 interviews, both parents were present and provided their insights into the DAMA process but it was considered as a single in-depth interview. Basic demographic data were also recorded and clinical profile of the child was extracted from hospital records. The details regarding the financial help to the patients were obtained from the records of the patient relation office (PRO) of the hospital.

The recordings were manually transcribed by one of the investigators. Each transcription underwent an additional review for accuracy by one of the investigators other than the original transcriber.

### 2.1. Statistical Analysis

Descriptive statistics [mean (SD), frequency (%)] were used to depict the clinical profile of the babies as well as sociodemographic profile of the parents. Chi square test was used to assess the association of discharge against medical advice with sociodemographic variables. All the quantitative analysis was performed using STATA (14.2). Qualitative analysis of the transcriptions was manually performed to understand various aspects of DAMA. Common threads emerging out of the in-depth interviews were elaborated.

The Institutional Ethics Committee approved the study.

## 3. Results

**Figure 1:**
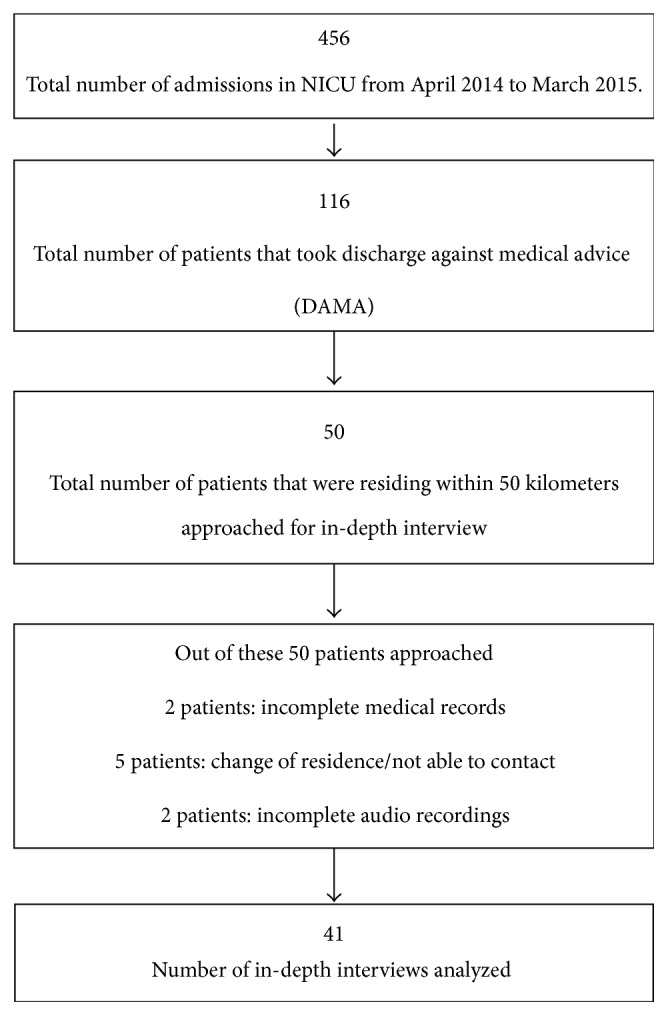
Flowchart of study population.

Out of the parents of 50 babies approached for in-depth interview, five changed their residence and two audio recordings were incomplete mainly due to nonwillingness of parents to talk about the reasons for discharge against medical advice. Further parents of two babies took discharge against medical advice almost immediately after the admission and hence the clinical and PRO related information was not available. Thus 41 in-depth interviews were completed and analyzed (Figure 1).

Although significantly more males (298) were admitted as compared to females (158), no difference in the proportion of males who were discharged against medical advice as compared to females (8.59% versus 7.60%, *p* = 0.7) was noted. Similar observation was noted for inborn versus outborn babies (7.1% versus 9.3%, *p* = 0.4) as well as BPL versus non-BPL categories (7.4% versus 8.8%, *p* = 0.6). One-third of the babies (34.1%) were discharged against medical advice on the first day of admission.

Sepsis, birth asphyxia, respiratory distress, and congenital heart disease were the most common diagnostic related groups in babies discharged against medical advice (Table 1). While nonaffordability emerged as the most common reason contributing to the decision of taking discharge against medical advice, inappropriate behavior of the staff (especially PRO staff) and feeling of hopelessness due to poor prognosis and current condition of the baby also contributed to the unwarranted decision. Lack of facilities for relatives was also reported by few parents (Table 2).

Theme based excerpts from the in-depth interviews are presented below in patients'/guardians' own words removing slang that was used occasionally.

### 3.1. Nonaffordability

*P- “That we have already told you. Money, nothing else. They asked for 10,000 rupees per day. How do you think we could pay that much! We don't have that much money.”*

*P- “Reduce cost. Keep discounts in bills. Quicken the treatment.”*

*P- “They told us that if you have money then continue the treatment. Otherwise, we cannot help it.”*

*I- “Were you willing to continue the treatment if you would have had money?”*

*P- “Yes, of course. We would definitely complete the treatment.”*

*I- “If you didn't have money issues then you would not have taken discharge?”*

*P- “Yes sir. On the first day, they told me that she had difficulty in breathing. On the 2nd day, they told me that she was not taking food. On the 3rd day, they said that they want to aspirate some water from the waist. The 4th day, she got an infection. They ordered one injection of 13,000 rupees. I can't spend that much money, so it was very difficult for me.”*

*I- “Did you go because they were increasing the hospital stay?” *

*P- “Yes and the charges were also more than I had expected.” *

*“Give money or go. They were talking like this.”*

*“We were told to keep the baby for so many days but budget was the issue.”*

*“They should understand the difference between rich and poor patients. Doctor will do his work but the hospital should only understand.”*

*P- “It was about money.”*

*P- “Everybody loves their children but the finances are a big problem, if we were rich we would have taken her to the best doctor.”*

### 3.2. Inappropriate Behavior of Staff

*P- “In medical (the term used to describe the hospital), they are only sucking money out of poor people. Money was the main reason. They want Talati's report, Sarpanch's report, etc. so we don't have it. Medical hospital only wants money. They didn't understand the condition of patients, especially the poor people.”*

*P- “You have to improve only in money matter. Give some advantage of Krupa* (a scheme that provides financial cover for hospitalization in return for a small subscription)* to the patient, so that patient can manage their problems.”*

*P- “They know the financial condition of the patient. We don't have BPL number. Even if I sell my 2 Bigha land, it wouldn't be enough.”*

*I- “When did they ask you to get Sarpanch's recommendation?”*

*P- “When we were getting discharge, at that time they told us to get the requisition. That is not necessary.” So I got angry. *

*P- “They didn't even let 500 rupees go. My bill was 16,500 and I had given 16,000 and even then they said it won't work, so I gave 500 and took the bill. If they do so then small family people would die. There is no BPL card, look at my house.”*

*P- “Your doctors asked me if we got a sign of sarpanch or not. I have to pay the money, why should I get his sign?”*

*I- “Do you know about an office in the hospital which might help you for money.”*

*P- “No, we were just told about the operation and operation cost.”*

*P- “There also they asked for money, and then they took us to the PRO office. They didn't help me with the issue. They didn't respond well. They filled up my form after 3-4 days of admission. That was a bitter experience. Then we were supposed to pay 17,000 rupees. We thought that we would go home. But they said that we have to pay the bill first.”*

*I- “Then what did you do?”*

*P- “Then I asked my boss. We work for them. So they gave us 5,000–6,000 and my wife goes to different bungalows for cleaning and all. They also gave us some money. So we arranged money in that way and we left.”*

*P- “Money. The child needed treatment and the doctors asked for money. I had no money left. They told us that we have to keep our child there for 10 days at least. But what about the money? Then I thought it's all about destiny. I trust “Dashama”* (a local deity)* a lot. I told her that “Maa, this is your child now. I cannot do anything else. You save the child now.” Then we came back. We expected some help from the PRO office as well but they didn't help me for that purpose.”*

*I- “What did the PRO office people tell you?”*

*P- “They told us that they would inform if anything will happen but didn't explain anything to us. The doctor also kept on asking for hundreds of things. Drugs and medicines and why have not they brought it yet? We didn't eat for days.”*

*Doctors and Nurses*

*P- “They didn't allow us to go and meet the baby. Doctors didn't tell us things properly. They said pay the money and keep the child in the NICU. Then I went to the pharmacy to buy the medicines and suddenly my mother called me that they are shifting the child to cardiac.”*

*I- “Didn't they inform you before doing anything?”*

*P- “No. They just said that bring money, bring medicines. We were going to give money anyways. But they didn't listen to that.” *

*P- “Yes but the confusion and the communication gap also played a role in that. We were not informed anything and the weight of the baby was also not improving even after 2-3 days. We kept the baby on the machine but the things were not improving the way they should. They kept us confused. They should inform us before doing anything.” *

*I- “Anything else?”*

*P- “Yes. My wife was kept in the ward near NICU. Many students would come and ask every half an hourly about personal life like when did you get married, how did you get married, how often do you have sexual relationship?”*

*P- “They should go out and see the condition of the relatives; they cry a lot and are harassed a lot by the staff. I would say the same to the doctors that there has to be a way of talking to the patients who come from a village and have no knowledge about anything. We know that even the nurses and doctors are drained with all the work all day. But they should at least talk to old people properly”*

*I- “So the behavior of the sisters was not good?”*

*P- “No, the doctor's behavior was also not good. When we were talking about the investigations, he got a bit rude. I did not expect this from a doctor!”*

*I- “What are your suggestion about doctors and nurses?”*

*P- “The sisters had multiple problems. They were not helpful.” *

### 3.3. Poor Prognosis

*“There was no reason of money. We are ready to pay any charges for the baby. If the problem did not occur during delivery, then it would all have been over in a good way. But his brain was not working. That would mean that if he had survived, its effect would have been life-long. Doctors tried their best.”*

*“Doctor told us that she drank amniotic fluid and her brain was not working so we took discharge. Otherwise the treatment was good. Doctor's behavior was also good. Every doctor came and explained to us very well.”*

### 3.4. Others (Related to Hospital Policies or Communication Gap between Doctors/Nurses and Parents/Guardians)

#### 3.4.1. No Guarantee

*“We said that give us guarantee for the life of the baby. We would not spend 2 to 5 lakhs, just like that.” *

*“We were told about a heart problem and that money would be needed. It would cost 5 lakhs, but what if the child does not become healthy? They said in 30 years, total of 3 operations were needed. That also was not guaranteed. There was no guarantee whether the operation would be successful or not. They did not take any responsibility.”*

#### 3.4.2. No Improvement

*“Her father said that we will take discharge because no good treatment was given for 8 days. I paid 35,000 rupees. Still they spoiled the case.” *

*I- “So is that the reason you left?”*

*P- “Yes. What is the point in being there if there is no improvement?”*

*I- “What did they say?”*

*P- “They said the weight has to increase, only after that they will discharge the baby. So we left by choice.”*

#### 3.4.3. Not Willing for Operation

*P- “They advised us to go for the operation. The baby was only 24 hours old. So I did not get him operated and took discharge.”*

#### 3.4.4. Demands to Bring Various Documents

*“In emergency, in night will I bring my child to the hospital or the BPL card?” *

*“In emergency, we couldn't remember bringing anything, we were in a rush.”*

#### 3.4.5. Not Allowing Seeing the Baby

*“They were good but once, the doctor told me that I can go and see my child one time in a day. But when I went there, he told me that I shouldn't come as frequently as I might infect my child! But I have to see my kid right?”*

#### 3.4.6. No Sleeping or Stay Arrangements for Relatives

*P- “There is no facility to sit outside and there is only one fan. Also there is no guest house or a facility to stay.” *

*I- “Which facility is not there for the guests who come to see the patient?”*

*P- “There is no place for the relatives to sit.”*

#### 3.4.7. Forced to Take DAMA by Paternal Grandfather

*I- “Why did you take the discharge?” *

*P- “I wanted to get the operation done but my in-laws were not ready. So we took discharge.”*

*In spite of Taking DAMA, Some of the Parents Praised the Hospital*

*“Doctors are good and staff is good.” *

*I- “Was there any problem in the behavior of sisters or doctor?”*

*P- “They were all good. They were talking very nicely.”*

*I- “Were they talking to you about the condition of the patient?”*

*P- “Yes, but not in much detail, but that was good. No problems with that. The staff there was very nice. They were very fast in treating and admission.”*

*I- “Do you have any other suggestion for the hospital?”*

*P- “It was a nice experience. Hospital is doing a good job, and overall a good experience.”*

*P- “I was told to take the child home at night. They had tried the maximum number of doses of the medicine, but convulsion didn't stop, so there was no point in continuing the treatment. Baby could die at any time after discharging her. Even if the convulsions had stopped, the baby would have had mental problem.”*

*“Before this we did not want to take the name of Shree Krishna Hospital but now it is fine. My experience here was fine. There is no charge. All things are free.”*

*I- “Did you have any problem with doctors or sister? Anyone misbehaved with you?”*

*P- “No, no, nothing like that.”*

*I- “Anything else?”*

*P- “The treatment was good. All facilities were good but the money was an issue. They should help us with financial issues. Doctors had done their best.”*

## 4. Discussion

This study noted high prevalence (25.4%) of DAMA in NICU. One-third of the babies (34.1%) were discharged against medical advice on the first day of admission. No gender bias was observed related to DAMA. Infections and asphyxia were the most common clinical conditions and affordability and prognosis were the most common reasons for babies discharged against medical advice.

A review of studies conducted in Iran revealed that the prevalence of DAMA varies from 4% to 35% in different clinical departments [14]. The DAMA rates are reported to be low (1% to 5%) in pediatric departments [6–11]. It appears that the study setting, sociocultural factors, time of study, and region have an impact on the DAMA rates. Although there is scarcity of such studies in neonates, all of these studies indicated that DAMA rates are higher in neonates. Interestingly, one-third of the patients were discharged against medical advice on the first day of admission. Similar observation was noted in other studies [7–13].

The current study revealed that infections and asphyxia were the most prevalent diagnoses. This finding corroborates with other studies in pediatric [7, 9, 11] as well as neonatal populations [12, 13].

Nonaffordability and feeling of hopelessness emerge as the main factors contributing to discharge against medical advice while serious communication gap has cascading effect as noted in studies conducted in developing economies [6–13]. In fact, few studies made a case for universal health coverage through National Insurance Scheme and improving counseling services.

From providers' perspective, patient's lack of insight, communication, mistrust, and conflict may lead to DAMA [15] but in general pediatricians showed empathy and positive attitude towards patients whose parents request discharge against medical advice [16]. Communication, informed consent, and underlying psychiatric issues are endorsed in practical management of DAMA [17]. In general, DAMA is a complex issue and the solutions must be sought considering sociocultural and environmental background.

While the Indian health system was working well in early years after independence, the focus on family planning in the late 70s destabilized it and emergence of private providers inflated the costs albeit with somewhat improved quality of care [18]. Admitting the disparity in healthcare in 2002 [19], Indian Government launched National Rural Health Mission in 2005 with many schemes to improve healthcare in India [20].

Catering specifically to newborns, home based and facility based newborn care models were introduced that were partially successful. For example, Bal Sakha (Child's Friend) scheme was launched in Gujarat in January 2009 to facilitate expert care to newborns. A fixed amount of about 30 USD was offered to take care of a baby. The study site immediately accepted the scheme but terminated the same due to huge losses incurred. A study conducted at the same site revealed that the average cost of hospitalization in children admitted to pediatric intensive care unit was about 200 USD [21]. At the minimum, the Bal Sakha scheme should have reimbursed the hospital on the basis of Diagnosis-Related Group (DRG) to make it sustainable.

To enhance community participation and access to the healthcare system, NRHM developed a workforce of community health worker, namely, Accredited Social Health Activist (ASHA). Home Based Newborn Care guidelines were released in 2011 and updated in 2014 [22]. ASHAs were trained in essential newborn care through modules 6 and 7 [23]. Unfortunately lack of monitoring, supportive supervision, and infrastructural facilities could not empower ASHAs sufficiently [24].

Indian health system needs a total revamp to ensure health coverage to all. It is possible through an integrated national health system focusing on public primary care system leveraging private sector in a regulated manner [25].

From hospital's perceptive, it is evident that there is some serious communication gap between the parents and staff especially the PRO executive. The possible reason could be isolated counseling by technical experts (physicians) and PRO executives. Creating adequate facilities for relatives is a minor administrative issue, ignored for a long time. Realizing the fact that NICU admission can ruin the budget of even middle class families, the study site started extending all the discounts available to BPL families, to those whose income is less than 10000 Indian rupees (~150 USD).

## 5. Limitations 

This is a single-center study and as evident from other studies the reasons for DAMA vary depending on sociocultural and environment issues. The efforts to prevent DAMA may not be generalizable beyond the region.

## 6. Conclusion

The issue of DAMA needs attention. Hospital policy reforms including proper and timely communication with empathy and sensitivity, orientation of PROs towards NICU, and enhanced infrastructural facilities for the attendants may be explored. The central and state governments should extend adequate financial support through flexible and carefully designed schemes catering to newborns.

## Figures and Tables

**Table 1:** Distribution of diagnosis in the study group (*N* = 41).

Diagnosis	*n*	Percent
Small for gestational age	11	26.8
Disseminated intravascular coagulation	5	12.2
Respiratory distress syndrome	7	17.1
Sepsis	21	51.2
Anemia	2	4.9
Shock	5	12.2
Meconium aspiration syndrome	2	4.9
Hypoxic ischaemic encephalopathy	19	46.3
Acute kidney injury	7	17.1
Meningitis	2	4.9
Pulmonary hemorrhage	2	4.9
Hyperbilirubinemia	3	7.3
Necrotising enterocolitis	1	2.4
Persistent pulmonary hypertension	3	7.3
Intracranial haemorrhage	1	2.4
Congenital heart disease	6	14.6

Note: some patients can have more than one diagnosis.

**Table 2:** Distribution of reasons for DAMA in the study group (*N* = 41).

Reasons for DAMA	*n*	Percent
Nonaffordability	29	70.7
Demands to bring various documents	1	2.4
Inappropriate behavior of doctors	3	7.3
Inappropriate behavior of nurses	4	9.8
Inappropriate behavior of PRO personnel	8	19.5
Poor prognosis	9	22.0
No guarantee	5	12.2
No improvement	11	26.8
Forced to take DAMA by paternal grandfather	1	2.4
No sleeping arrangements for relatives	1	2.4
No stay arrangements for relatives	1	2.4
Not allowing seeing the child	1	2.4
Not willing for surgery on their newborn child	1	2.4

Note: some patients can have more than one reason for DAMA.

## Appendix

### The Interview Guide

The interview was conducted based on the following themes:

1. Background information about the family covering the economic, educational, professional, social, and medical aspects.
2. Do you remember that baby?
3. What was the problem with the baby?
4. To which hospital was the baby taken and how?
5. What happened to the baby at the hospital?
6. What was your experience at the hospital?
7. How was the baby discharged?
  - Why had the baby taken DAMA?
  - What could have prevented DAMA?
8. What happened to the baby afterwards?
9. Do you still consider that taking DAMA was in the best interest of the baby?
10. What are your suggestions?

## Abbreviations

DAMA: Discharge against medical advice
PRO: Patient relation office
ICU: Intensive care unit
NICU: Neonatal intensive care unit
I: Interviewer
P: Patient
BPL: Below poverty line
LSCS: Lower segment cesarean section
LBW: Low birth weight
VLBW: Very low birth weight
ELBW: Extremely low birth weight
Dr.: Doctor
No.: Number
Freq: Frequency
OPD: Outpatient department.
